# Cumulative Incidence and Predictors of Progression in Corticosteroid-Naïve Patients with Sarcoidosis

**DOI:** 10.1371/journal.pone.0143371

**Published:** 2015-11-17

**Authors:** Yusuke Inoue, Naoki Inui, Dai Hashimoto, Noriyuki Enomoto, Tomoyuki Fujisawa, Yutaro Nakamura, Takafumi Suda

**Affiliations:** 1 Second Division, Department of Internal Medicine, Hamamatsu University School of Medicine, Hamamatsu, Japan; 2 Department of Clinical Pharmacology and Therapeutics, Hamamatsu University School of Medicine, Hamamatsu, Japan; Medical School Hannover, GERMANY

## Abstract

**Background:**

Assessment of the clinical course of sarcoidosis requires long-term observation. However, the appropriate period of follow-up for sarcoidosis remains unclear, especially in patients without indication of corticosteroid therapy at the time of diagnosis.

**Objective:**

This study aimed to clarify the cumulative incidence and identify risk factors for disease progression in corticosteroid-naïve sarcoidosis patients.

**Methods:**

The clinical courses of 150 Japanese patients with sarcoidosis, who were followed for more than 2 years and had no indication for corticosteroid therapy at diagnosis, were retrospectively reviewed. Disease progression was defined as worsening of pulmonary sarcoidosis, development of new organ involvement, or extrapulmonary organ damage. The cumulative incidence of progression was estimated by generating a cumulative incidence curve with the Fine and Gray method.

**Results:**

The median follow-up duration was 7.7 years (interquartile range, 4.7–13.6 years). Thirty-two (21%) patients experienced disease progression. New organ involvement appeared in 16 patients (11%). The 6-month, and 1-, 5-, 10-, and 15-year cumulative incidence of progression was 2%, 5%, 15%, 28%, and 31%, respectively. The number of organs involved at diagnosis was an independent predictor for progression with a multifactorial adjusted hazard ratio of 1.71 (95% confidence interval, 1.11–2.62). The optimal cut-off of the number of organs involved at diagnosis to identify future progression was three.

**Conclusions:**

In corticosteroid-naïve sarcoidosis patients, the risks of disease progression are comparable from 0–5 years and 5–10 years after diagnosis. The number of organs involved at diagnosis is a useful predictor for progression of sarcoidosis.

## Introduction

Sarcoidosis is a systemic granulomatous disorder of unknown cause. Sarcoidosis affects multiple organs, and patients with sarcoidosis have a diverse clinical course and prognosis. Spontaneous remission occurs in two thirds of cases [1]. On the other hand, disease progression occurs in some fraction of cases of sarcoidosis. The prevalence of deterioration of pulmonary sarcoidosis widely ranges from 13% to 75% of patients [2–5]. Although assessment of disease progression in sarcoidosis is a major problem, in which patients and in what manner it occurs are not fully understood. Furthermore, few data are available concerning the long-term course of sarcoidosis because most studies assessed the natural history of this disease 2–5 years after diagnosis [3, 4, 6–8].

There is little evidence regarding the duration in which patients with sarcoidosis need to be followed. Some studies have proposed that patients with sarcoidosis should be monitored for at least 3 years after termination of corticosteroid therapy, and no more follow-up is necessary unless new or worsening symptoms occur or extrapulmonary lesions are involved [2, 9]. In contrast to patients in whom corticosteroid therapy has successfully ended, patients with persistent disease should be followed for a long time, sometimes for all of their lives [9]. For sarcoidosis patients in whom there is no indication for corticosteroid therapy at the time of diagnosis, no consensus has been made on the adequate duration of follow-up. This is because studies on the clinical course of sarcoidosis included patients receiving corticosteroid [2–8, 10, 11] and there is a lack of information on the natural history focusing on corticosteroid-naïve patients.

Therefore, in the present study, we aimed to examine the clinical course in patients with sarcoidosis who did not require treatment with corticosteroid at the time of diagnosis. In addition, we examined risk factors for progression of sarcoidosis in these patients.

## Materials and Methods

### Study subjects

A total of 199 consecutive patients who were diagnosed as having sarcoidosis between March 1990 and September 2012 at Hamamatsu University Hospital, Japan, were reviewed. The diagnosis and assessment of organ involvement were based on the American Thoracic Society, the European Respiratory Society, and the World Association of Sarcoidosis and Other Granulomatous Disorders consensus statement [12], as well as the Diagnostic Standard and Guideline for Sarcoidosis determined by the Japan Society of Sarcoidosis and Other Granulomatous Disorders (JSSOG) [13]. At the time of diagnosis, all of the patients underwent comprehensive assessment of clinical symptoms, a physical examination, a laboratory test, and an imaging study. Bronchoalveolar lavage fluid (BAL) and transbronchial lung biopsy were performed as previously described [14]. To detect extrapulmonary involvement, patients routinely underwent electrocardiography (ECG) and ophthalmological assessments. Gallium scanning, echocardiography, Holter ECG, cardiac magnetic resonance imaging (MRI), abdominal ultrasound, thallium scintigraphy, and ^18^fluorodeoxy glucose-positron emission tomography (FDG-PET) were performed at the discretion of physicians.

Among 199 diagnosed patients, 36 patients who were followed less than 2 years were excluded from this study. Furthermore, 13 patients who had corticosteroid therapy initiated at the time of diagnosis were excluded. After the exclusion of these 49 patients, 150 patients were included in this study. The study population was ethnically homogeneous, comprising only Japanese patients. They were followed every 3–6 months in the outpatient setting, and the disease state was assessed by a physical examination, chest radiography, pulmonary function tests, ECG, and serum chemistry including calcium, liver enzymes, creatinine, and angiotensin-converting enzyme (ACE). Chest/abdominal computed tomography (CT), scintigraphy, echocardiography, Holter ECG, cardiac/abdominal/muscle MRI, abdominal ultrasound, FDG-PET, or an ophthalmologic examination was performed when disease progression or development of new organ involvement was suspected. All of the patients underwent more than two consecutive measurements, and outcomes were evaluated using assessments at different time points. The study was approved by the Institutional Review Board of Hamamatsu University School of Medicine (certificate #14–078). Because of the retrospective manner of the study, written consent from participants for use of records was waived. All patients’ records/information were anonymized and de-identified prior to analysis. This information was notified on the website (http://hamamatsu-lung.com/study.html).

### Data collection

Clinical, radiological, and laboratory data, including BAL and pathological information, were retrospectively obtained from paper and electronic medical records. The number of involved organs was counted according to the definition used in the Case Control Etiologic Study of Sarcoidosis (ACCESS) Research Group [15] and the Diagnostic Standard and Guideline by JSSOG [13]. Involvement of mediastinal lymph nodes was included in lymph node lesions, separate from lung involvement. Hypercalcemia and hypercalciuria were excluded from counting of affected organs.

### Definition of outcomes

The clinical course was classified into the following three groups using previously reported criteria [4, 16]. (1) Progression was defined as (a) deterioration of pulmonary sarcoidosis, which was classified as a decrease in forced vital capacity (FVC) and forced expiratory volume in the first second (FEV_1_) >10% from baseline, a decrease in FVC or FEV_1_ >10% from baseline and a worsening of any of pulmonary symptoms, or worsening pulmonary infiltrates as detected on chest radiography with worsening of pulmonary symptoms; (b) development of new organ involvement; or (c) worsening of extrapulmonary lesions that required systemic corticosteroid therapy. Pulmonary symptoms needed to be present for more than 1 month. (2) Improvement was defined as (a) an increase in FVC and FEV_1_ of >10% from baseline without worsening pulmonary symptoms, (b) an increase in FVC or FEV_1_ of >10% from baseline with improvement of pulmonary symptoms, or (c) improvement of indicated pulmonary infiltrates without worsening of pulmonary symptoms. (3) All other patients were defined as stability.

### Statistical analysis

Cumulative incidence curves were used to estimate the cumulative risk of progression of sarcoidosis using the method of Fine and Gray [17], and comparison was performed by Gray’s test. Any death not caused by sarcoidosis was considered as a competing risk in the analysis. Time to progression was calculated as the time from the date of a diagnosis of sarcoidosis to the date of occurrence of deterioration. The outcome was censored if a patient had not worsened by the time of the last follow-up or if a patient was lost to follow-up. Fine–Gray’s proportional hazards model was used to examine predictors of disease progression after adjustment for age and radiographic stage at baseline, both of which are known to be adverse prognostic factors in sarcoidosis [4, 9, 12, 18, 19]. The receiver operating characteristic (ROC) curve was used to determine an appropriate cut-off number of organs involved at the time of diagnosis to predict occurrence of progression. The most suitable cut-off point was chosen as that which combined maximal sensitivity with the best specificity. Statistical analyses were performed using the software EZR (Saitama Medical Center, Jichi Medical University, Saitama, Japan), which is a graphical user interface for R (The R Foundation for Statistical Computing, Vienna, Austria, version 3.0.2) [20]. More precisely, EZR is a modified version of R commander (version 2.0–3) that was designed to add statistical functions that are frequently used in biostatistics. A value of *P* < 0.05 was considered to be significant.

## Results

### Characteristics of sarcoidosis patients at the time of diagnosis

A total of 150 sarcoidosis patients were included in this study. Table 1 shows the baseline characteristics of patients. The median age at diagnosis was 51 years (interquartile range [IQR], 32–61 years) and 103 patients (69%) were women. Eighty-three (55%) patients had general, pulmonary, or ophthalmological symptoms. At initial assessment, 133 (89%) patients underwent gallium scanning, 105 (70%) echocardiography, 86 (57%) Holter ECG, 45 (30%) cardiac MRI, 39 (26%) abdominal ultrasound, 35 (23%) thallium scintigraphy, and 7 (5%) FDG-PET. The median number of affected organs was three (IQR, 2–3; range, 1–7). Involved organs that were detected at initial assessment are shown in Table 2. Almost all of the patients (n = 148, 99%) had lung lesions (ACCESS proposed assessment instrument: definite, n = 143) and were divided into four groups based on findings on chest radiography: 10 patients had a normal radiograph, 81 patients had bilateral hilar lymphadenopathy (BHL) alone, 45 patients had BHL and lung parenchymal involvement, and 14 patients had lung parenchymal involvement alone. None of the patients had lung fibrosis at the time of diagnosis. Only two patients had the acute type of sarcoidosis and no patients had Lofgren’s syndrome. At initial assessment, 143 patients had mediastinal lymph node involvement. The other common extrathoracic lesion was ocular (n = 86, 57%: ACCESS proposed assessment instrument: definite, n = 85). Skin lesions were found in 28 patients (ACCESS proposed assessment instrument: definite, n = 27) in whom erythema nodosum was diagnosed in three patients. Serum ACE levels were elevated in 49 (33%) patients. FVC, FVC % predicted, and the FEV_1_/FVC ratio were within the normal range in most patients.

**Table 1:** Characteristics of patients with sarcoidosis at the time of diagnosis.

Characteristics	Total (n = 150)
Age, years	51 (32–61)
Sex, male/female	47/103
Smoking status, ever/never	57/93
BMI, kg/m^2^	21.7 (19.8–24.4)
Symptoms, yes/no	83/67
Number of affected organs	3 (2–3)
Histological diagnosis, yes/no	119/31
Serum ACE, IU/L^a^	18.5 (14.6–23.3)
Radiographic stage	
0/I/II/III/IV	10/81/45/14/0
Pulmonary function tests^b^	
FVC, L	2.77 (2.34–3.47)
FVC, % predicted	95.4 (86.2–106.9)
FEV_1_, L	2.26 (1.83–2.93)
FEV_1_/FVC, %	81.5 (76.1–86.5)
Bronchoalveolar lavage^c^	
Total cells, ×10^5^/mL	1.4 (0.9–2.4)
Lymphocytes, %^d^	9.9 (6.2–19.5)
CD4/CD8 ratio	5.3 (2.7–9.1)

BMI, body mass index; ACE, angiotensin-converting enzyme; FVC, forced vital capacity; FEV_1_, forced expiratory volume in 1 second. Radiographic stages 0, I, II, III, and IV represent a normal appearance, bilateral hilar lymphadenopathy (BHL) alone, BHL and lung parenchymal involvement, lung parenchymal involvement without BHL, and pulmonary fibrosis, respectively.

Data are presented as median (interquartile range) or number of patients.

^a^ The upper limit of normal level, 21.4 IU/L.

^b^ Values for FVC, FVC % predicted, FEV_1_, and the FEV_1_/FVC ratio were missing in 10 patients.

^c^ Because of extremely low recovery rates and missing data of BAL analyses, values for total cells, lymphocytes, and the CD4/CD8 ratio were not available in six, two, and six patients, respectively.

^d^ The upper limit of normal level, 17% [21].

**Table 2:** Organ involvement that was detected at the time of diagnosis.

Affected organs	Number	%
Lungs	148	98.7
Lymph nodes	144	96.0
Mediastinal lymph nodes	143	95.3
Extrathoracic lymph nodes	28	18.7
Eyes	86	57.3
Skin	28	18.7
Parotid/salivary gland	13	8.7
Muscle	11	7.3
Spleen	7	4.7
Thyroid	3	2.0
Neurosarcoidosis	2	1.3
Bone/joint	2	1.3
Heart	1	0.7
Liver	1	0.7
Kidney	1	0.7

### Cumulative incidence of progression of sarcoidosis

**Fig 1:**
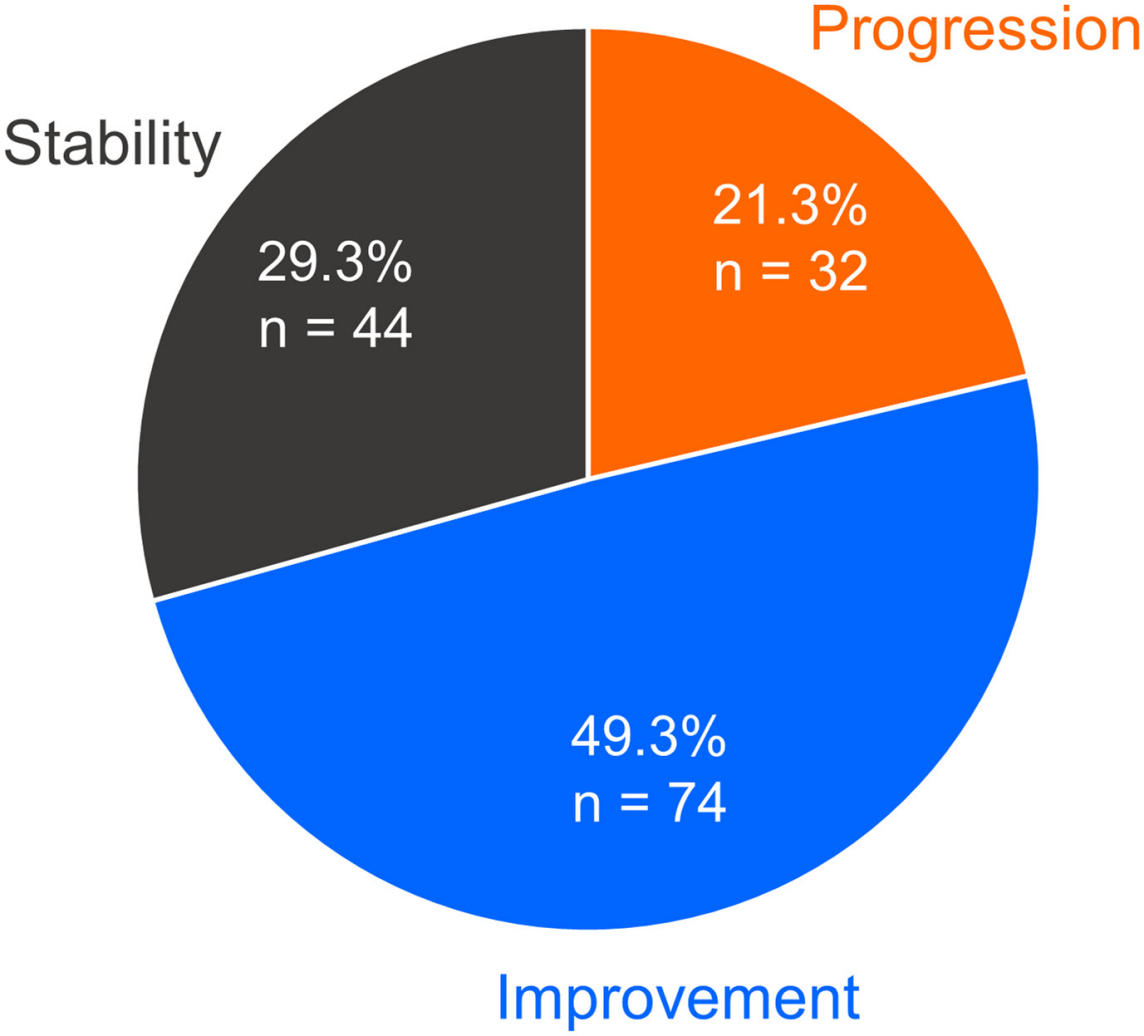
Proportion of clinical outcomes observed in sarcoidosis patients. Outcomes that were classified into three groups are depicted in a pie chart. Of 150 patients, 21.3% (n = 32) experienced disease progression. Improvement and stability of disease were observed in 74 (49.3%) and 44 (29.3%) patients, respectively.

**Fig 2:**
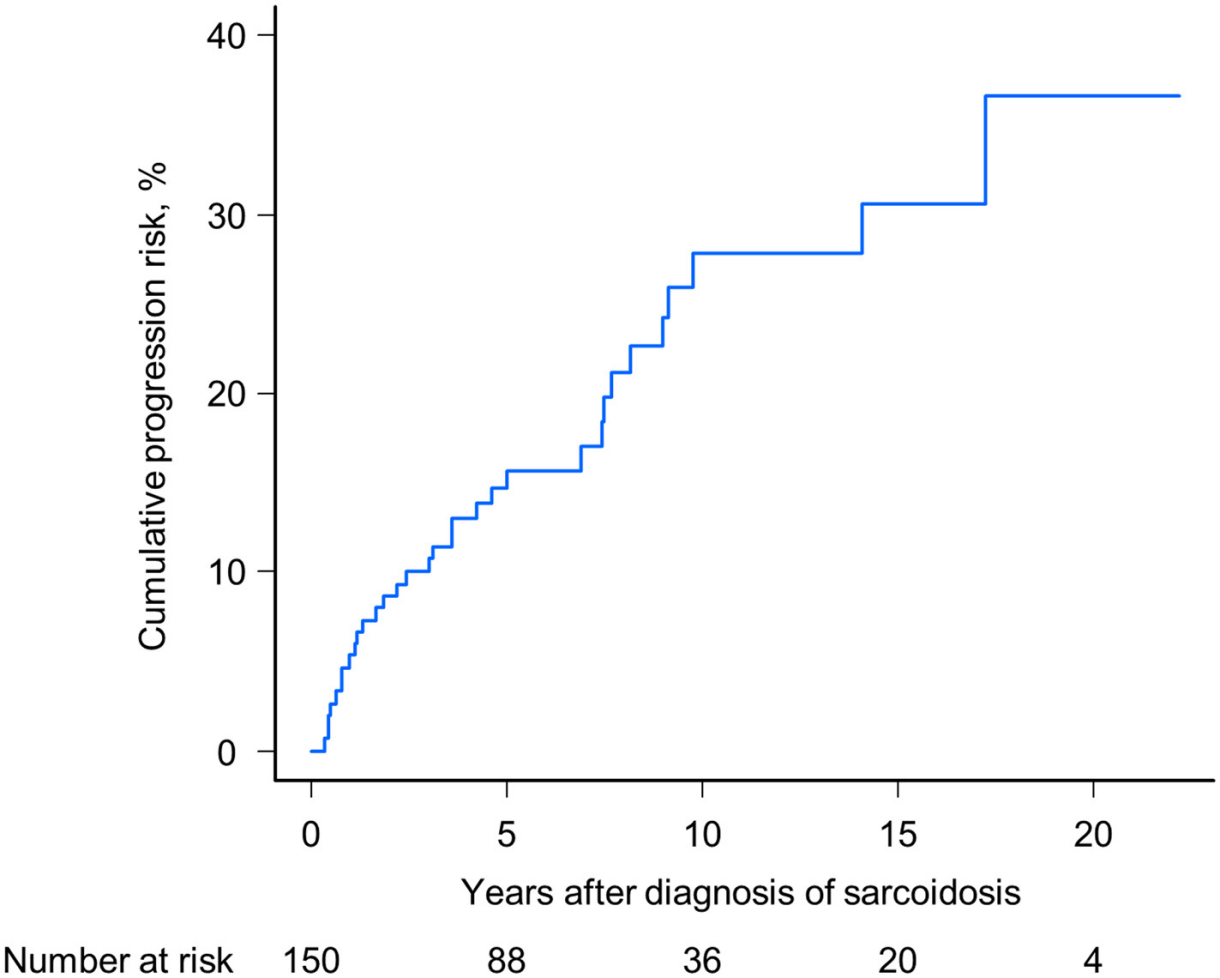
Cumulative incidence of disease progression in corticosteroid-naïve patients with sarcoidosis. The blue line represents the estimation curve of cumulative incidence.

We evaluated the clinical course of sarcoidosis patients with a median follow-up of 7.7 years and an IQR of 4.7–13.6 years. The numbers of patients who were classified into the categories of progression, improvement, and stability were 32 (21%), 74 (49%), and 44 (29%), respectively (Fig 1). In the progression group, the numbers of patients who met each defined criterion of “a” and “b” were 15 (47%) and 14 (44%), allowing for overlap of criteria “a” and “b” in one patient. Four patients required systemic corticosteroid therapy because of worsening of extrapulmonary lesions during follow-up (13%, criterion of “c”), all of whom had deterioration of ocular lesions. Of the 32 patients whose disease had progressed, corticosteroid therapy was initiated in 18 (56%) patients. The starting dose of prednisolone was 30–60 mg per day and was gradually reduced. The median duration of corticosteroid therapy was 2.4 years (IQR, 1.6–7.6 years). The cumulative incidence of disease progression at 6 months, and 1, 5, 10, and 15 years after the time of diagnosis was 2% (95% confidence interval [CI], 0.5–5.3%), 5% (95% CI, 2.1–8.9%), 15% (95% CI, 9.4–21.1%), 28% (95% CI, 18.8–37.6%), and 31% (95% CI, 20.4–41.3%), respectively (Fig 2).

### Effect of the number of organs involved on progression of sarcoidosis

**Fig 3:**
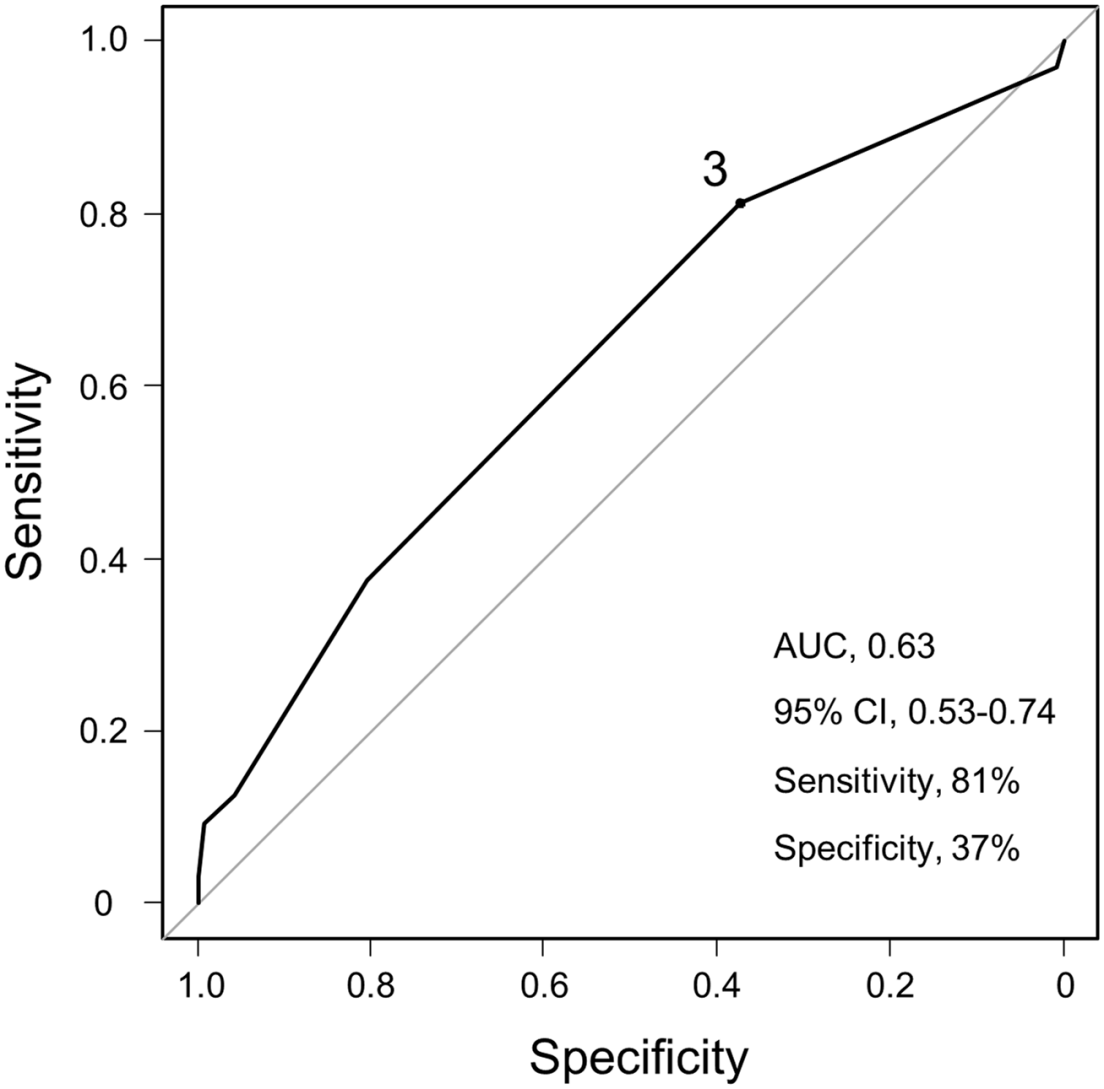
Optimal cut-off number of organ involvement at initial assessment to identify future progression. Receiver operating characteristic curve analysis for evaluating the value of the baseline number of organ involvement for predicting progression of sarcoidosis.

**Fig 4:**
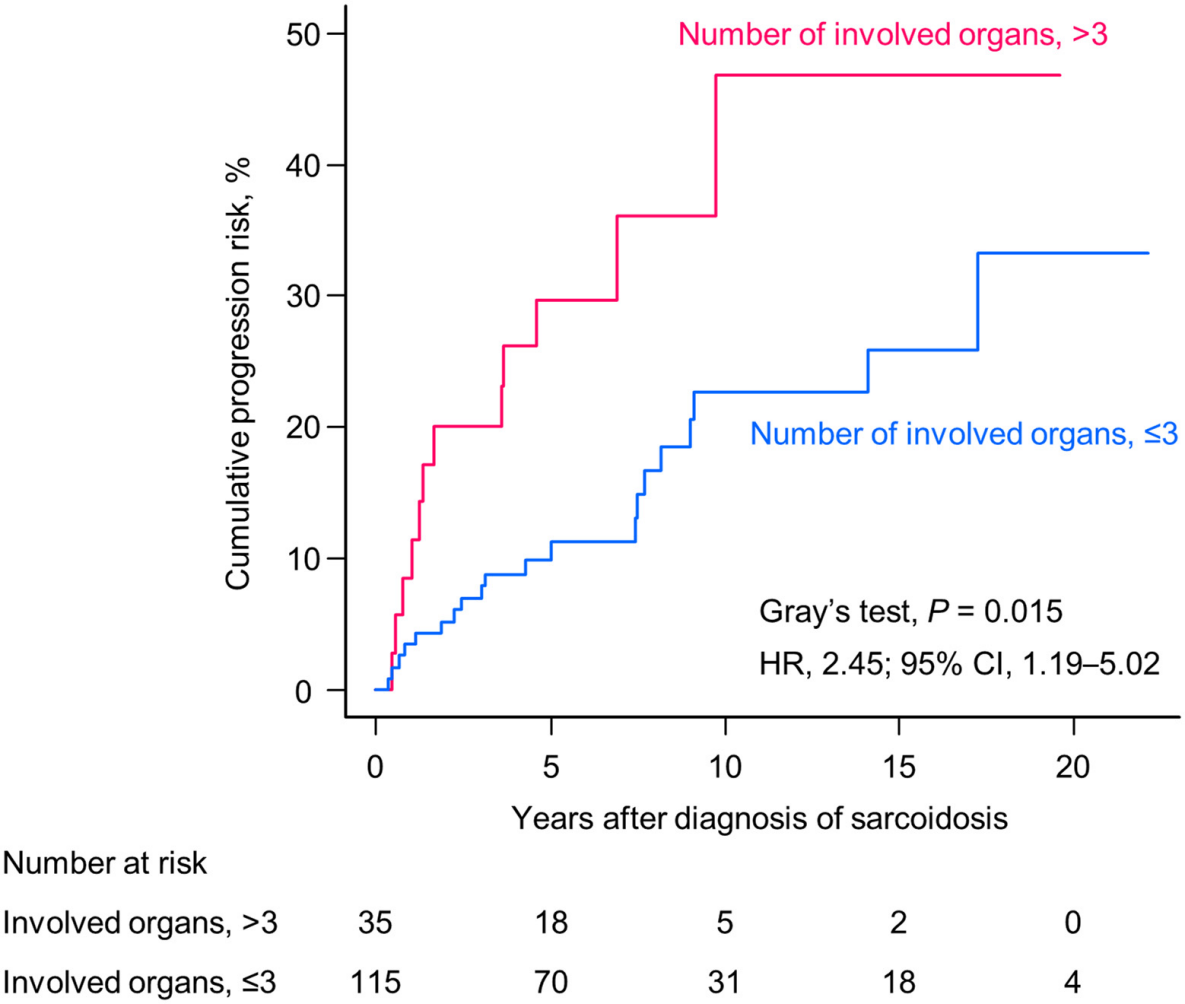
Estimates for the cumulative risk of progression of sarcoidosis according to organ involvement. A significant difference was observed in patients with more than three affected organs at initial assessment (pink line) compared with those who had three or less affected organs (blue line).

In multivariate analysis, the number of organs involved at diagnosis was an independent significant predictor of disease progression (hazard ratio [HR], 1.71; 95% CI, 1.11–2.62; *P* = 0.015; Table 3) after adjusting for age (per 10-year increase) and radiographic stage at baseline. To evaluate the predictive value of the number of organs involved at diagnosis for future deterioration, ROC analysis was performed (Fig 3). The area under the curve was 0.63. A cut-off value of three was determined with a sensitivity and specificity of 81% and 37%, respectively. According to this cut-off value, we performed subgroup analysis for the cumulative incidence of disease progression. The cumulative risk of progression was significantly higher in patients who had more than three affected organs (HR, 2.45; 95% CI, 1.19–5.02; *P* = 0.015 by Gray’s test; Fig 4).

**Table 3:** Hazard ratios for factors that are potentially associated with progression of sarcoidosis.

Characteristics	Per unit for HR	Univariate HR	95% CI	*P* value	Multivariate HR	95% CI	*P* value
Age	10 years	1.03	0.81–1.29	0.84	0.92	0.69–1.23	0.58
Sex	Male/female	0.92	0.43–1.97	0.83			
BMI	1 kg/m^2^	1.06	0.96–1.17	0.28			
Symptoms	Yes/no	1.71	0.84–3.50	0.14			
Histological diagnosis	Yes/no	1.91	0.69–5.33	0.22			
Radiographic stage	One stage	1.15	0.72–1.84	0.56	0.92	0.52–1.61	0.76
Number of organs involved	One organ	1.63	1.15–2.29	0.0058	1.71	1.11–2.62	0.015
Serum ACE	≥21.4/<21.4, IU/L	1.35	0.65–2.77	0.42			
BAL							
Total cells	10^4^/mL	1.00	0.99–1.01	0.87			
Lymphocytes	≥17/<17, %	0.99	0.47–2.07	0.98			
CD4/CD8 ratio	≥3.5/<3.5	1.42	0.64–3.14	0.38			
Pulmonary function tests							
FVC % predicted	≥80/<80, %	1.75	0.43–7.09	0.43			
FEV_1_/FVC	≥70/<70, %	1.40	0.36–5.51	0.63			

BMI, body mass index; ACE, angiotensin-converting enzyme; FVC, forced vital capacity; FEV_1_, forced expiratory volume in 1 second; HR, hazard ratio; CI, confidence interval.

### Cumulative incidence of new organ involvement

**Fig 5:**
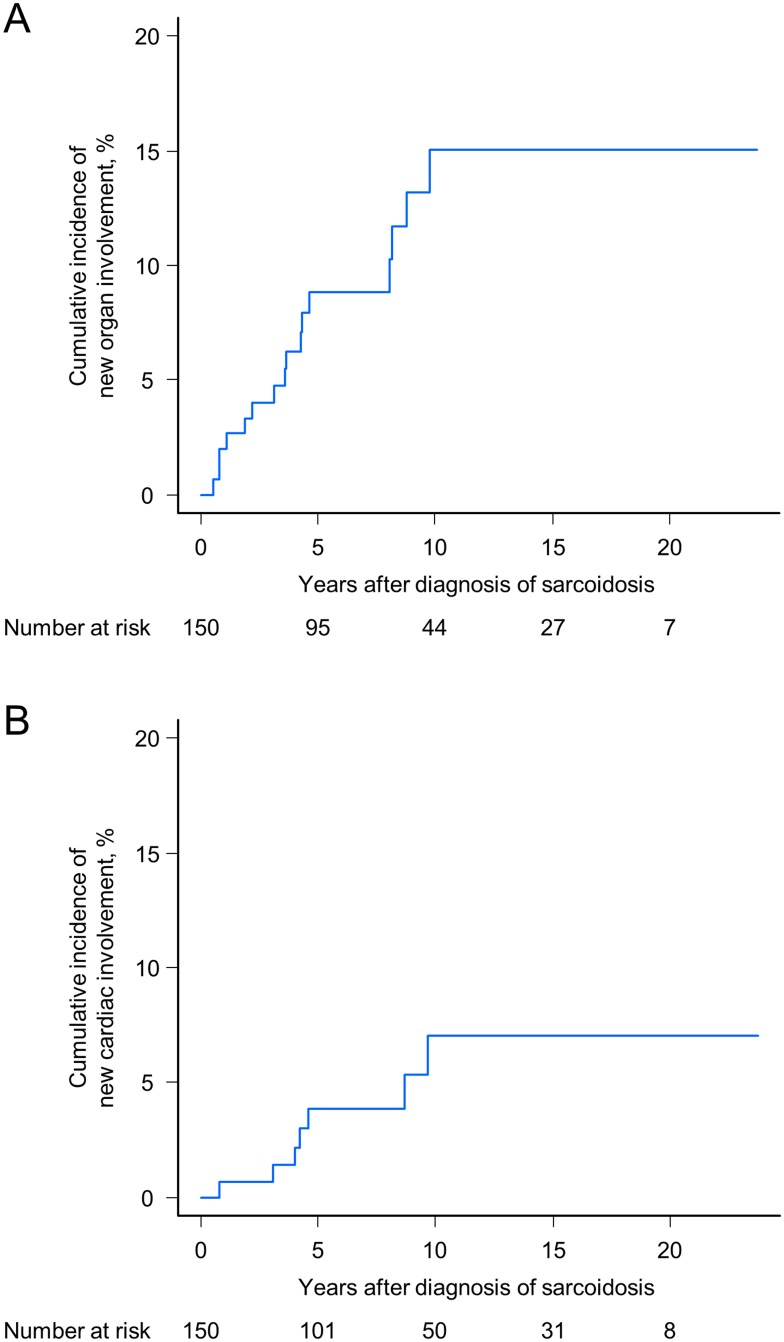
Cumulative incidence of any new organ involvement (A) and new cardiac involvement (B). The blue lines represent the estimation curves of cumulative incidence.

During the entire follow-up period, new organ involvement appeared in 16 (11%) patients, including two patients who were initially classified as progression of pulmonary sarcoidosis. Involved organs were the heart (n = 7), skin (n = 7), superficial lymph nodes (n = 5), spleen (n = 3), kidney (n = 3), intra-abdominal lymph nodes (n = 2), parotid/salivary gland (n = 2), liver (n = 1), bone (n = 1), muscle (n = 1), and tonsils (n = 1). The cumulative incidence of any new organ involvement at 6 months after diagnosis of sarcoidosis was 0%, and 2% (95% CI, 0.5–5.3%) at 1 year, 9% (95% CI, 4.8–14.4%) at 5 years, 15% (95% CI, 8.5–23.2%) at 10 years, and 15% (95% CI, 8.5–23.2%) at 15 years (Fig 5A). With regard to the cardiac involvement, arrhythmia occurred in six patients (complete atrioventricular block, n = 2; ventricular tachycardia, n = 2; sinus arrest and intermittent ventricular tachycardia, n = 1; atrial fibrillation, n = 1). One patient presented with massive pericardial effusion due to cardiac sarcoidosis. Systemic corticosteroid therapy was initiated in four patients, and three patients required pacemaker insertion. When restricted to new cardiac involvement, the cumulative risks at 6 months, and 1, 5, 10, and 15 years after diagnosis were 0%, 0.7% (95% CI, 0.1–3.4%), 4% (95% CI, 1.4–8.3%), 7% (95% CI, 2.9–13.7%), and 7% (95% CI, 2.9–13.7%), respectively (Fig 5B). Similar to the cumulative risk of entire disease progression, the cumulative incidence of any new organ involvement and that of new cardiac involvement in the first 5 years and the next 5 years were almost comparable. Interestingly, no new organ involvement occurred as an initial progression event 10 years after the diagnosis of sarcoidosis.

## Discussion

To the best of our knowledge, this study is the first to investigate the cumulative incidence of disease progression and new organ involvement in corticosteroid-naïve patients with sarcoidosis. The risks of initial deterioration at 0–5 and 5–10 years after diagnosis were almost equivalent. We found that the number of involved organs at the time of diagnosis was an independent factor to predict disease progression.

In sarcoidosis, 10–30% of patients are generally considered as chronic or progressive in their clinical course [12]. However, there is no consensus on the definition of progression and most reports focused on outcomes of pulmonary sarcoidosis [4, 16, 18]. The definition of deterioration of sarcoidosis proposed by Hunninghake et al. [4] included a worsening of symptoms and a decrease in pulmonary function. Huggins et al. [16] included worsening of pulmonary symptoms and at least one of the following two criteria: a decrease in FVC and/or FEV_1_; and/or worsening pulmonary infiltrates. In the present study, we used these definitions to determine progression of sarcoidosis, and added the two criteria of occurrence of new organ involvement and worsening of extrapulmonary lesions. The criteria of progression used in this study appear to be more appropriate than those used in other studies because they are likely to correspond with situations in which corticosteroid therapy is considered.

Previous studies usually included patients who received corticosteroid therapy owing to disease severity and involved organ damage [2–8, 10, 11]. In contrast, the long-term clinical course of patients with sarcoidosis who do not receive corticosteroid therapy has not been fully evaluated, which raises the clinical question of how long these patients should be followed up. Therefore, the present study assessed disease progression, focusing on patients who had no indication for systemic corticosteroid therapy at the time of diagnosis. We estimated that the proportion of patients who had disease progression during a long follow-up time was 21% with the median follow-up duration of 7.7 years. Patients who were monitored for longer than 2 years were included in this study because disease persisting for more than 2 years is often considered as chronic sarcoidosis [5].

Several risk factors for progression of sarcoidosis have been reported, such as black race [3, 10], a longer disease duration [11], female sex [3], older age [4, 9, 12, 18], extrapulmonary lesions [2], advanced radiological stage [4, 9, 12, 19], and treatment with corticosteroids [3, 11]. The cumulative probability of persistence of disease activity was described in a cohort of Spain including 193 patients using the Kaplan–Meier method [22]. In this previous study, the persistence of disease activity was defined as the presence of at least one of the following parameters: clinical features; chest radiographic abnormalities; a decline in spirometry; increased serum calcium levels; increased serum ACE levels; increased gallium uptake; alveolitis in BAL analysis; and positive biopsy. Using statistical methodology that was similar to our study, the study identified the absence of erythema nodosum, pulmonary infiltrate on chest radiography, splenomegaly, age ≥40 years, and the absence of lymphadenopathy in chest radiography as independent risk factors [22]. In the present study, the number of organs involved was the sole predictive factor of progression, and a cut-off value of three was determined. It should be noted that involvement of mediastinal lymph nodes was counted as lymph node lesions according to the ACCESS tool and the Diagnostic Standard and Guideline for Sarcoidois proposed by JSSOG [13]. Chest radiographic stage, which is known to be a useful prognostic marker of pulmonary sarcoidosis, was not identified as the prognosis predictor in our univariate and multivariate analyses. The clinical course of sarcoidosis is heterogeneous and variable [12], and it greatly differs in included patients, area, race, the prevalence of acute sarcoidosis, and endpoints. In particular, the difference in definitions of outcomes between studies should be noted. We defined disease progression as not only deterioration of pulmonary sarcoidosis, but also development of new organ involvement or worsening of extrapulmonary lesions that required systemic corticosteroid therapy. In addition, because we studied corticosteroid-naïve patients, we were able to assess the natural history of sarcoidosis without the confounding influence of corticosteroid therapy.

Sarcoidosis is a systemic disorder that affects multiple organs. During the follow-up period of this study, the heart and skin were the most frequently observed organs with newly developed lesions of sarcoidosis. In the ACCESS study [6], new organ involvement was reported to occur in 23% of sarcoidosis patients at a 2-year follow-up, among whom the most frequent site was the skin, whereas the heart was the fifth most common. Cardiac sarcoidosis is increasingly recognized as a cause of heart failure, arrhythmia, and sudden death. In Japan, nearly 80% of sarcoidosis-related deaths are due to cardiac involvement [23]. Because cardiac lesions are difficult to detect, partly because of their focal nature [24], manifestation of cardiac sarcoidosis that was not detected at the time of initial examination has been the focus of attention. In the present study, new cardiac involvement occurred in seven patients. The cumulative risks at 5 and 10 years after diagnosis were 4% and 7%, respectively. This finding indicated that the cumulative incidence of new cardiac involvement in the first 5 years was comparable with that in the second 5 years. Another finding of the current study was that no patients developed new organ lesions as an initial deterioration event over 10 years after diagnosis. These results of the present study might help physicians to decide to continue or stop follow-up of patients whose disease is stable for a long time.

There are several limitations in this study. First, this was a single-institution, uncontrolled, retrospective study. The follow-up time and intervals of examinations were dependent on physicians and patients. However, follow-up of all sarcoidosis patients without symptoms and treatment for a long time in a prospective manner according to protocols are not realistic. Second, all of the patients who were included in this study were Japanese. Ethnic and genetic factors can influence the clinical course and prognosis of sarcoidosis [12, 25]. Third, this study assessed only the initial events to estimate the cumulative incidence of progression. Therefore, no conclusion can be drawn concerning the appropriate follow-up duration in patients who experienced deterioration of disease once. Fourth, tools to diagnose and identify organ involvement have been added and changed. In the present study, we mainly used the ACCESS tool [15], not an updated tool [26]. Different examinations were used in the present study depending on the time of diagnosis, which might have caused bias in characterizing our cohort. Finally, the criteria of disease progression in this study did not include information on diffusing capacity for carbon monoxide (DL_CO_). Although DL_CO_ is a sensitive detector of lung impairment [27, 28], DL_CO_ values could not be included in the analyses because of the small number of patients in whom DL_CO_ values were available in our cohort.

In conclusion, we assessed the cumulative incidence of progression in corticosteroid-naïve sarcoidosis patients and identified the number of organs involved at the time of diagnosis as an independent predictor. Although there is currently no consensus on the duration to follow patients with sarcoidosis, our results suggest that the appropriate follow-up time for corticosteroid-naïve sarcoidosis patients is at least 10 years. Further studies with larger numbers are required to confirm our results and to determine the optimal follow-up period that is tailored to each patient’s individual risk.

## References

[1] Iannuzzi MC, Rybicki BA, Teirstein AS. Sarcoidosis. N Engl J Med. 2007;357: 2153–2165. 18032765 10.1056/NEJMra071714

[2] Rizzato G, Montemurro L, Colombo P. The late follow-up of chronic sarcoid patients previously treated with corticosteroids. Sarcoidosis Vasc Diffuse Lung Dis. 1998;15: 52–58. 9572002

[3] Gottlieb JE, Israel HL, Steiner RM, Triolo J, Patrick H. Outcome in sarcoidosis. The relationship of relapse to corticosteroid therapy. Chest. 1997;111: 623–631. 9118698 10.1378/chest.111.3.623

[4] Hunninghake CW, Gilbert S, Pueringer R, Dayton C, Floerchinger C, Helmers R, et al Outcome of the Treatment for Sarcoidosis. Am J Respir Crit Care Med. 1994;149: 893–898. 8143052 10.1164/ajrccm.149.4.8143052

[5] Johns CJ, Schonfeld SA, Scott PP, Zachary JB, MacGregor MI. Longitudinal study of chronic sarcoidosis with low-dose maintenance corticosteroid therapy. Outcome and complications. Ann N Y Acad Sci. 1986;465: 702–712. 3460404 10.1111/j.1749-6632.1986.tb18549.x

[6] Judson MA, Baughman RP, Thompson BW, Teirstein AS, Terrin ML, Rossman MD, et al Two year prognosis of sarcoidosis: the ACCESS experience. Sarcoidosis Vasc Diffuse Lung Dis. 2003;20: 204–211. 14620163

[7] Pietinalho A, Ohmichi M, Lӧfroos AB, Hiraga Y, Selroos O. The prognosis of pulmonary sarcoidosis in Finland and Hokkaido, Japan. A comparative five-year study of biopsy-proven cases. Sarcoidosis Vasc Diffuse Lung Dis. 2000;17: 158–166. 10957764

[8] Baughman RP, Lower EE. Features of sarcoidosis associated with chronic disease. Sarcoidosis Vasc Diffuse Lung Dis. 2015;31: 275–281. 25591138

[9] Costabel U. Sarcoidosis: clinical update. Eur Respir J. 2001;32: 56s–68s. 11816825

[10] Israel HL, Karlin P, Menduke H, DeLisser OG. Factors affecting outcome of sarcoidosis. Influence of race, extrathoracic involvement, and initial radiologic lung lesions. Ann N Y Acad Sci. 1986;465: 609–618. 3460398 10.1111/j.1749-6632.1986.tb18537.x

[11] Rodrigues SC, Rocha NA, Lima MS, Arakaki JS, Coletta EN, Ferreira RG, et al Factor analysis of sarcoidosis phenotypes at two referral centers in Brazil. Sarcoidosis Vasc Diffuse Lung Dis. 2011;28: 34–43. 21796889

[12] Statement on sarcoidosis. Joint Statement of the American Thoracic Society (ATS), the European Respiratory Society (ERS) and the World Association of Sarcoidosis and Other Granulomatous Disorders (WASOG) adopted by the ATS Board of Directors and by the ERS Executive Committee, February 1999. Am J Respir Crit Care Med. 1999;160: 736–755. 10430755 10.1164/ajrccm.160.2.ats4-99

[13] The Japanese Society of Sarcoidosis and Other Granulomatous Disorders (JSSOG). Diagnostic standard and guideline for sarcoidosis-2006. Jpn J Sarcoidosis and Granulomatous Disorders. 2007;27: 89–102.

[14] Inui N, Chida K, Suda T, Nakamura H. TH1/TH2 and TC1/TC2 profiles in peripheral blood and bronchoalveolar lavage fluid cells in pulmonary sarcoidosis. J Allergy Clin Immunol 2001;107: 337–344. 11174202 10.1067/mai.2001.112273

[15] Judson MA, Baughman RP, Teirstein AS, Terrin ML, Yeager H, the ACCESS Research group. Defining organ involvement in sarcoidosis: the ACCESS proposed instrument. ACCESS Research Group. A Case Control Etiologic Study of Sarcoidosis. Sarcoidosis Vasc Diffuse Lung Dis. 1999;16: 75–86. 10207945

[16] Huggins JT, Doelken P, Sahn SA, King L, Judson MA. Pleural effusions in a series of 181 outpatients with sarcoidosis. Chest. 2006;129: 1599–1604. 16778281 10.1378/chest.129.6.1599

[17] Fine JP, Gray RJ. A proportional hazards model for the subdistribution of a competing risk. J Am Stat Assoc. 1999;94: 496–509.

[18] Panselinas E, Judson MA. Acute pulmonary exacerbations of sarcoidosis. Chest. 2012;142: 827–836. 10.1378/chest.12-1060 23032450

[19] Reich JM, Johnson RE. Course and prognosis of sarcoidosis in a nonreferral setting. Analysis of 86 patients observed for 10 years. Am J Med. 1985;78: 61–67. 3966490 10.1016/0002-9343(85)90463-2

[20] Kanda Y. Investigation of the freely available easy-to-use software 'EZR' for medical statistics. Bone Marrow Transplant. 2013;48: 452–458. 10.1038/bmt.2012.244 23208313 PMC3590441

[21] Warr GA, Martin RR, Holleman CL, Criswell BS. Classification of bronchial lymphocytes from nonsmokers and smokers. Am Rev Respir Dis. 1976;113: 96–100. 1082283 10.1164/arrd.1976.113.1.96

[22] Mañá J, Salazar A, Manresa F. Clinical factors predicting persistence of activity in sarcoidosis: a multivariate analysis of 193 cases. Respiration. 1994;61: 219–225. 7973108 10.1159/000196341

[23] Iwai K, Sekiguti M, Hosoda Y, DeRemee RA, Tazelaar HD, Sharma OP, et al Racial difference in cardiac sarcoidosis incidence observed at autopsy. Sarcoidosis. 1994;11: 26–31. 8036339

[24] Silverman KJ, Hutchins GM, Bulkley BH. Cardiac sarcoid: a clinicopathologic study of 84 unselected patients with systemic sarcoidosis. Circulation. 1978;58: 1204–1211. 709777 10.1161/01.cir.58.6.1204

[25] Mirsaeidi M, Machado RF, Schraufnagel D, Sweiss NJ, Baughman RP. Racial difference in sarcoidosis mortality in the United States. Chest. 2015;147: 438–449. 10.1378/chest.14-1120 25188873 PMC4314818

[26] Judson MA, Costabel U, Drent M, Wells A, Maier L, Koth L, et al The WASOG Sarcoidosis Organ Assessment Instrument: An update of a previous clinical tool. Sarcoidosis Vasc Diffuse Lung Dis. 2014;31: 19–27. 24751450

[27] Subramanian I, Flaherty K, Martinez F. Pulmonary function testing in sarcoidosis In: Baughman RP, ed. Sarcoidosis. New York: Taylor and Francis Group; 2006;210: 415–433.

[28] Boros PW, Enright PL, Quanjer PH, Borsboom GJJM, Wesolowski SP, Hyatt RE. Impaired lung compliance and *D*L,CO but no restrictive ventilatory defect in sarcoidosis. Eur Respir J. 2010;36: 1315–1322. 10.1183/09031936.00166809 20378598

